# Les corps étrangers laryngo-trachéo-bronchiques: expérience de l'hôpital d'instruction des armées Omar Bongo Ondimba (HIAOBO) de Libreville

**DOI:** 10.11604/pamj.2015.20.298.4576

**Published:** 2015-03-26

**Authors:** Adèle-Rose Ngo Nyeki, Jérôme Miloundja, Asmaou Bouba Dalil, Jean Marcel Mandji Lawson, Sylvie Nzenze, Emery Sougou, Annie Nziengui, Léon N'zouba

**Affiliations:** 1 Service Oto-rhino-laryngologie et Chirurgie Cervico-Faciale, Hôpital d'Instruction des Armées Omar Bongo Ondimba, Libreville, Gabon; 2 Service d'Anesthésie et Réanimation, Hôpital d'Instruction des Armées Omar Bongo Ondimba, Libreville, Gabon

**Keywords:** Corps étrangers, laryngo-trachéo-bronchique, endoscopie, urgence, foreign body, Laryngo-tracheobronchial, endoscopy, emergency

## Abstract

L'inhalation accidentelle de corps étranger est fréquente chez l'enfant et exceptionnelle chez l'adulte. Elle représente une urgence respiratoire pouvant mettre en jeu le pronostic vital. L'objectif était de présenter les difficultés de prise en charge des corps étrangers laryngo-trachéo-bronchiques (CELTB). Il s'agissait d'une étude rétrospective réalisée sur une période de 6 ans (Avril 2006-Mars 2012), dans les services d'Oto-Rhino-Laryngologie et de chirurgie cervico-faciale (ORL-CCF) de l'HIA OBO de Libreville. Nous avons répertorié 21 dossiers de patients admis pour corps étrangers laryngo-trachéo-bronchiques. Leur âge moyen était de 8,95 ans avec des extrêmes de 3 et 37 ans. Les enfants représentaient 90% de cas. Le sex-ratio était de 2,30. Les corps étrangers étaient à 55% d'origine alimentaire et à 45% d'origine métallique. Leur localisation était laryngée dans 60% des cas, bronchique dans 35% et trachéale dans 5% des cas. Sur le plan clinique, la toux était retrouvée chez tous les patients. Il existait un syndrome de pénétration dans 60% de cas. La découverte était fortuite lors d'un syndrome de séjour broncho-pulmonaire dans 30% des cas. L'extraction des corps étrangers était réalisée par voie endoscopique et sous anesthésie générale. Chez 47,6% de cas, nous avons effectué une trachéotomie première. Les suites opératoires étaient favorables dans 95,24% et un décès a été noté. La prise en charge des CELTB doit être précoce et nécessite une parfaite collaboration entre anesthésistes et chirurgiens. Leur extraction se fait par voie endoscopique d'où l'intérêt de disposer, dans un service d'ORL-CCF, de matériel endoscopique adapté à l’âge.

## Introduction

Les corps étrangers laryngo-trachéo-bronchiques (CELTB) sont fréquents chez les enfants dès l’âge de 6 mois [1] et exceptionnels chez l'adulte. Leur inhalation survient accidentellement et ils constituent une urgence respiratoire pouvant mettre en jeu le pronostic vital. Leur prise en charge a bénéficié des progrès de l'endoscopie et de la réanimation notamment en milieu pédiatrique. L'objectif de ce travail était de rapporter les cas de corps étrangers laryngo-trachéo-bronchiques admis dans le service d'Oto-rhino-laryngologie et de Chirurgie cervico-faciale (ORL-CCF) de l'Hôpital d'Instruction des Armées Omar Bongo Ondimba (HIAOBO) de Libreville et de présenter les difficultés liées à leur prise en charge dans notre contexte d'exercice.

## Méthodes

Nous avons effectué une étude rétrospective sur une période de 6 ans allant d'Avril 2006 à Mars 2012 dans les services d'ORL et de Chirurgie cervico-faciale de l'HIAOBO de Libreville. Nous avons colligé tous les dossiers de patients ayant été pris en charge pour CELTB. Pour chaque dossier, nous avons étudié les aspects épidémiologiques, les délais de consultation et de séjour du CELTB, les signes cliniques et radiologiques, la localisation et la nature des CELTB et enfin, les modalités thérapeutiques et évolutives.

## Résultats

### Sur le plan épidémiologique

**Figure 1:**
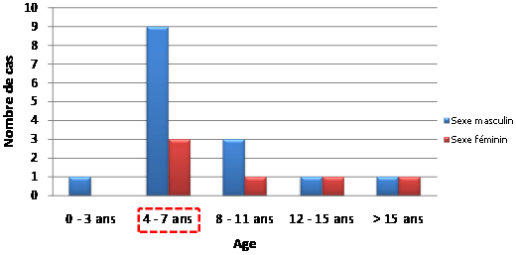
Répartition des patients selon l’âge et le sexe

La population de notre étude comprenait 21 patients. Durant la période de l’étude, 1049 interventions chirurgicales sous anesthésie générale ont été réalisées dont 123 cas d'extraction de corps étrangers des voies aérodigestives supérieures (VADS). L'extraction des CELTB a donc représenté 2% de notre activité chirurgicale et 17% des corps étrangers des VADS. L’âge moyen de nos patients était de 8,65 ans avec des extrêmes de 3 ans et 37 ans. Il y avait 19 enfants et 3 adultes. Treize cas avaient un âge compris entre 3 et 6 ans, soit 62% de notre série. Les enfants dont l’âge moyen était de 6,4 ans représentaient 90% de notre échantillon. Nous avons eu 15 hommes et 6 femmes, soit un sex ratio de 2,5. La Figure 1 illustre la répartition de notre échantillon selon l’âge et le sexe.

### Sur le plan clinique

**Figure 2:**
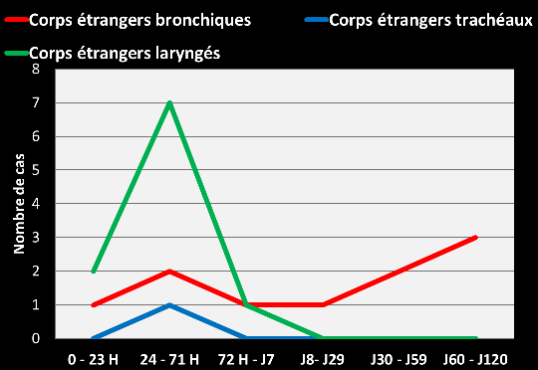
Délai de séjour des corps étrangers dans les voies respiratoires

Le délai d’évolution entre l'inhalation du corps étranger et la consultation était compris entre une heure et 48 heures pour 55% des patients, entre 3 jours et 30 jours pour 25% et au-delà de 30 jours pour 25% des patients. Le délai moyen de séjour des corps étrangers était de 1 jour dans la trachée, 2 jours dans le larynx et de 56 jours dans les bronches, soit une moyenne globale de 19,66 jours (Figure 2). La toux était présente chez tous les patients. Le syndrome de pénétration a été retrouvé chez 60% des patients; 30% avaient un syndrome de séjour broncho-pulmonaire et 10% une détresse respiratoire.

### Au plan radiologique

**Figure 3:**
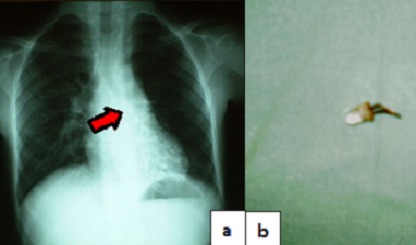
Corps étrangers bronchique gauche(a), néon de radio-transistor, inhalé depuis 90 jours et révélé par une bronchopneumonie gauche(b)

**Figure 4:**
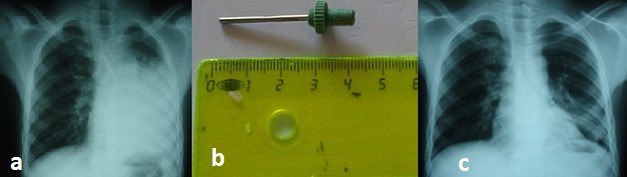
Corps étrangers bronchiques avec atélectasie gauche (a), tige de pompe à ballon inhalée depuis 2 jours (b), normalisation du poumon gauche 5 jours après extraction du corps étranger (c)

La radiographie cervico thoracique était normale dans 50% des cas. Dans 45% des cas, les corps étrangers étaient radio opaques. Les corps étrangers étaient localisés dans le larynx dans 50% des cas, dans les bronches dans 45% des cas et dans la trachée dans 5% des cas. En ce qui concerne la localisation bronchique, la bronche souche droite était la plus concernée avec 78% des cas. Nous avons noté 30% de cas de bronchopneumonie (Figure 3) et 10% de cas d'atélectasie pulmonaire (Figure 4).

### Nature des corps étrangers

Les corps étrangers étaient d'origine alimentaire dans 57,14% de cas et d'origine non alimentaires dans 42,86% de cas dont 28,57% des cas par des fragments métalliques de jouets (Tableau 1).

**Tableau 1:** Nature des corps étrangers (n = 21)

Nature des corps étrangers (CE)	Effectif	%
**CE d'origine alimentaire**	**12**	**57,14%**
Arêtes et os alimentaires	9	42,86%
Cacahuètes	3	14,28%
**CE d'origine non alimentaire**	**9**	**42,86%**
Fragments métalliques de jouets	6	28,57%
Vis	1	4,76%
Ampoules	1	4,76%
Perles	1	4,76%

### Sur le plan thérapeutique

Une endoscopie sous anesthésie générale a été effectuée chez tous les patients. Il s'agissait d'une laryngoscopie chez 50% des cas, les corps étrangers étant laryngés et d'une bronchoscopie pour les autres patients. Dix patients, soit 47,6% de notre série, ont eu une trachéotomie première pour permettre l'extraction des corps étrangers à l'aide d'une optique d'endoscopie de 0°. Il s'agissait des patients porteurs de corps étrangers dans la trachée ou dans les bronches. Le délai moyen entre la trachéotomie et la décanulation de ces patients était de 1,34 jours. Les suites opératoires ont été simples chez 20 patients, soit 95,24% des cas. Nous avons noté un cas de décès, soit 4,76%. Ce décès est survenu par œdème sous glottique sévère après extraction d'un corps étranger sous glottique incarcéré, sans trachéotomie de sécurité.

## Discussion

### Age

L'inhalation d'un corps étranger est un accident domestique fréquent chez l'enfant. Il représente 90,5% de cas dans notre série, avec une moyenne d’âge de 6,4 ans. Ces données sont similaires à celles retrouvées dans la littérature africaine [2–4]. Il est classique de décrire deux pics de fréquence: Le premier pic correspond à la période de la préhension, c′est-à-dire dès 6 mois avec un maximum au cours de la deuxième année. C'est la période d'exploration de l'environnement par l'enfant avec sa bouche qu'il utilise comme une « troisième main ». De plus, l'absence de dents postérieures et l'impossibilité de mastiquer facilitent l'inhalation des corps étrangers qui se retrouvent en entier dans les voies aériennes; Le deuxième pic se situe autour de 6 ans, et correspond à l’âge où les enfants deviennent de « petits bricoleurs ». Par ailleurs, c'est une période de relâchement de la surveillance des enfants par l'entourage, les exposant ainsi aux accidents domestiques.

Chez l'adulte, il s'agit d'un accident rare qui survient le plus souvent au décours d'un repas comme pour 3 de nos patients; le corps étranger étant souvent localisé au niveau du larynx.

### Sexe

Le sexe masculin est plus atteint avec un sex-ratio de 2,5. La plupart des auteurs s'accordent sur ce constat: 2,42 pour Diop et al [4] et 2 pour Vokwely et al [5]. Ceci s'explique par le fait que les petits garçons, en général, ont un instinct de découverte plus développé et sont plus turbulents que les petites filles du même âge.

### Délai de consultation

Le délai de consultation est variable. Plus de la moitié, soit 55% des patients ont étés vus en consultation durant les 2 premiers jours qui suivaient l'inhalation du corps étrangers comme le rapporte Ouoba et al [6]. Mais dans d'autres études, les délais de consultations sont plus longs notamment dans celle de Sissokho et al [3] où 69% des patients étaient vus après les 48 premières heures et celle de Watanabe et al [7] qui rapportent une moyenne de 90 jours. Ces retards de consultation sont dus à plusieurs facteurs qui tiennent à la nature fugace du syndrome de pénétration qui peut passer inaperçu, aux difficultés diagnostiques et à l'absence de services spécialisés dans certaines régions de notre pays. Ces patients vont alors multiplier des consultations dans des centres de santé avant d’être reçu dans un hôpital de référence.

### Délai de séjour des corps étrangers

Le délai moyen de séjour des corps étrangers dans les voies respiratoires dans notre échantillon est de 19,66 jours, il est plus long pour les corps étrangers à localisation bronchique. Des délais similaires sont retrouvés dans la littérature [8] surtout pour les localisations bronchiques lorsque le syndrome de pénétration n'a pas été identifié. Dans ce contexte, le corps étranger reste enclavé dans une bronche et se manifeste par des signes de bronchopneumopathie récidivante ou chronique.

### Syndrome de pénétration

Il a été retrouvé dans 60% des cas, un syndrome de pénétration à l'interrogatoire ou à l'examen clinique des patients. Dans la littérature sa fréquence varie entre 23 et 83,7% [3, 6, 8]. Il se manifeste par un accès brutal de suffocation accompagné d'une toux expulsive aboyante. C'est le syndrome pathognomonique d'inhalation qui est souvent sous-estimé et dont l’évaluation dans la littérature est variable. En effet, l'entourage peut être absent lors de la crise et ne pas le rapporter lors de l'interrogatoire; de plus, ce syndrome est souvent absent dans certaines localisations notamment au niveau de la margelle laryngée.

### Bilan radiologique

Les radiographiques cervico thoraciques sont souvent normales, comme pour Sissokho et al [3], qui en ont retrouvé 59%. Ceci est dû à la radio transparence de certains corps étrangers notamment alimentaires. En effet, une radiographie normale n'exclut pas la présence de CELTB. Des images radiologiques de bronchopneumopathie, d'atélectasie ou de dilatation de bronche constituent des signes évocateurs. Ils témoignent souvent d'un séjour prolongé du corps étrangers dans les bronches entrainant une réaction inflammatoire notamment pour les corps étrangers organiques [9]. Dans notre série, 77,7% des corps étrangers bronchiques sont retrouvés dans la bronche souche droite, comme pour la plupart des auteurs [3, 5]. L'existence d'un angle trachéo-bronchique plus obtus à droite qu’à gauche constitue la principale explication.

### Nature du corps étranger

Les corps étrangers de notre série sont surtout de nature alimentaire ou organique comme dans la littérature [2–6]. Avant 3 ans, les corps étrangers sont le plus souvent d'origine alimentaires puisque l'enfant découvre son environnement et sa bouche constitue une zone d'exploration; et dès l’âge de 3 ans, les corps étrangers deviennent métalliques ou plastiques et sont souvent des fragments de jouets ou de tout autre objet comme c'est le cas dans notre étude.

### Prise en charge

Le traitement repose sur l'extraction du corps étranger par voie endoscopique [1, 3, 6, 9]. Le maître de la broncho œsophagoscopie, Chevalier Jackson, disait en 1951: « Tout corps étranger des voies digestives ou aériennes qui a pénétré par les voies digestives ou aériennes doit être extrait par les même voies à condition qu'il n'ait migré au travers de la paroi perforée de ces voies » [10]. Nous avons effectué chez tous nos patients une extraction par voie endoscopique sous anesthésie générale. Cependant, en cas d’échec d'extraction par voie endoscopique, en présence de corps étranger vulnérant ou de perforations, la voie externe de cervicotomie ou de thoracotomie peut être indiquée [10].

### Place de la trachéotomie

Dans notre étude, les patients qui avaient des corps étrangers trachéaux ou bronchiques ont eu une trachéotomie première per endoscopique constituant en même temps une voie d'intubation et d'accès au corps étranger. Certains auteurs ont effectué des trachéotomies premières avant l'extraction des corps étrangers devant une urgence respiratoire telle que la dyspnée laryngée majeure ou après l'extraction du corps étranger pour des raisons de sécurité afin de prévenir un œdème sous glottique suffocant [4, 11]. Cependant, cet acte prolonge l'intervention et augmente la morbidité post opératoire [1, 4, 11]. Dans notre contexte, la trachéotomie a été effectuée pour pallier le manque de matériel adapté notamment des bronchoscopes pédiatriques. Cette trachéotomie permet de créer une ouverture trachéale haute et d'insérer une optique de 0°, d'un diamètre de 4 mm permettant de visualiser le corps étranger et de l'extraire à l'aide de micro pinces à mors. La trachéotomie est gardée en place moins de 48 heures pour permettre une surveillance et prévenir une détresse respiratoire secondaire à un œdème laryngé.

**Sur le plan évolutif** Nous avons eu un décès, soit 4,76% de notre échantillon. Ce décès est survenu dans les 6 heures qui ont suivi la laryngoscopie par un œdème sous glottique sévère qui aurait pu être prévenu par une trachéotomie de sécurité. Dans la littérature, le taux de décès est du même ordre de fréquence que dans notre série. Les auteurs estiment que les retards de prise en charge, l’œdème laryngé ou pulmonaire post opératoire, les insuffisances du plateau technique et de la surveillance clinique post opératoires sont les principales causes de décès [6, 8, 11].

## Conclusion

Les corps étrangers laryngo-trachéo-bronchiques sont fréquents chez les enfants de sexe masculin. Ils peuvent passer inaperçus et se manifester par des infections broncho-pulmonaires résistantes à tout traitement. Ils constituent une véritable urgence et peuvent engager le pronostic vital, chez les enfants et les adultes. Leur prise en charge doit être rapide et nécessite une parfaite collaboration entre anesthésistes et chirurgiens otorhinolaryngologistes. L'extraction de ces corps étrangers doit se faire par voie endoscopique d'où l'intérêt de disposer de matériel endoscopique pédiatrique dans les services d'ORL-CCF, voire de pédiatrie.
